# Hospitalizations of nursing homes residents: the role of reimbursement policies

**DOI:** 10.1186/2045-4015-3-3

**Published:** 2014-01-23

**Authors:** Toni Ashton

**Affiliations:** 1 School of Population Health, University of Auckland, Private Bag 92019, Auckland, New Zealand

## Abstract

Factors which contribute to hospitalization of nursing home residents are multiple and complex. They include dimensions of the policy setting as well as patient management practices. In seeking interventions to reduce such hospitalizations, most research has focussed on changing patient management. But provider reimbursement policies may also influence the decision to hospitalize nursing home residents. This includes payments to hospitals and general practitioners as well as to the nursing homes themselves. Differences in payment methods may mean that interventions that work well in one setting do not work well in another.

This is a commentary on http://www.ijhpr.org/content/3/1/2/.

## Commentary

The paper by Wagman et al. entitled “Variables correlated with elderly referral from nursing homes to general hospital” is the latest in a series of papers published over the past few years which have attempted to identify predictors of hospitalization by residents of nursing homes [1]. While some researchers have reported significant relationships between factors such as patient characteristics, staffing levels, advanced care planning, the characteristics of nursing homes, and the use of nurse practitioners, other authors, including Wagman et al., have found few or no significant relationships [2-5].

In the absence of clear evidence about the factors which contribute to high rates of hospitalization, the jury also remains out in terms of recommendations for appropriate interventions or policies for reducing these hospitalizations. Interventions which show some potential to reduce hospitalizations include increasing skilled staffing and improving the transition of patients from one health care setting to another [2]. However, a recent randomized controlled trial undertaken in New Zealand, which tested the impact of a package of evidence-based interventions on patient outcomes, found no impact on the number of acute hospital admissions or mortality [6].

To date, the focus of attention has been primarily on methods of improving patient management, including strengthening preventive practices. Yet the New Zealand study suggests that interventions that work in one setting may not work in another. In particular, efforts to reduce hospital referrals may be influenced by the prevailing reimbursement policies. Such policies may not encourage – and may actively discourage – efforts to reduce hospitalizations of residents by changing the way that patients are managed.

Only a few studies have focussed on the effect of reimbursement policies on hospitalization rates and most of these have been in the US. Intrator et al. found that the odds of hospitalization were lower in those states where Medicaid payments for nursing home residents were higher, while hospitalization odds were higher in states with bed-hold policies (i.e. where nursing homes are paid a retainer for a limited period while a resident is in hospital) [7]. Spector et al. [8] likewise found that bed-hold policies were associated with higher hospitalization rates but only for unavoidable conditions. The same study also found lower rates of avoidable hospitalizations where Medicaid payments were case-mix adjusted. Other studies have reported higher hospitalization rates for Medicaid patients (for whom reimbursement rates are usually lower than for other patients) and lower hospitalization rates for residents of not-for-profit nursing homes compared with residents of for-profit homes [9].

All of these studies have focussed only on nursing home reimbursement. But methods of payment for hospitals and for any health professionals who provide ambulatory care to nursing home residents may also impact on the incentive or disincentive to hospitalize.

I will use New Zealand to illustrate the types and directions of financial incentives that are likely to be associated with different types of payment systems. In New Zealand, nursing homes (which are called rest homes and which provide long-term care to patients who do not require 24-hour nursing care) are paid on a per day basis by District Health Boards (DHBs). Unlike the USA, the reimbursement rate is the same for both subsidized and non-subsidized patients. Thus there is no incentive for nursing homes to hospitalize based upon source of payment. However, because nursing home reimbursement is not case-mix adjusted, there may be some incentive to hospitalize more dependent patients whose care is more costly. Further, like the USA and Israel, bed-hold policies apply in which nursing homes are paid to hold the bed for up to a maximum of 3 weeks. While research studies suggest that this increases hospitalization, there are strong arguments in favour of bed-hold policies. The payment covers the fixed costs that must still be covered by nursing homes during the short-term absences of their residents. It also avoids the discharge of residents from what they may regard as their permanent home, and encourages continuity of care and of relationships between the facility, the resident and their family.

Wagman et al.’s study suggests that the availability and reimbursement of community-based primary medical services also need to be considered [1]. In New Zealand, these services are usually provided to nursing home residents by general practitioners (GPs), with provision of these services being part of the contractual arrangements between nursing homes and the DHBs. Methods of payment by nursing homes to GPs vary but usually include at least some fee-for-service component, especially where services are provided outside of normal working hours. Nursing homes therefore have an incentive to refer residents to a hospital emergency department (where services are provided free of charge) in preference to calling a GP. The funding of X-rays and other imaging services similarly provides an incentive for referral of residents to hospital because, while these services are provided free of charge through secondary care, the nursing home and/or the resident must pay a fee if they are accessed through primary care services.

For residents who are enrolled with a particular GP, the GP will also receive a monthly capitation payment based primarily upon the average cost of people aged 65 years and over. If GP consultation rates by nursing home residents are higher than the population average, or if the length of these consultations is longer, then GPs may avoid enrolling these patients. An additional problem is that, if a different GP sees an enrolled patient, then the charge for this consultation is clawed back from the GP with whom the patient is enrolled. If this occurs regularly, a GP may lose money on nursing home residents. Both of these effects may inhibit access to GP services by nursing home residents, and again, the probability of hospitalization may be increased [1,4,5].

While nursing homes in New Zealand appear to have some incentive to hospitalize patients, the opposite is true for general hospitals. Acute public hospital services are reimbursed by capped prospective budgets based upon estimated costs and volumes for each department. Increasing acute admissions therefore does not result in additional revenue for the hospital. Moreover, during hospital stays the DHBs - which own and manage the public hospitals - must pay both the cost of the hospital stay and the nursing home costs of subsidized residents during the bed-hold period. Thus hospitals have some incentive *not* to admit nursing home residents, especially subsidized residents, and to reduce the length of stay of residents who are admitted.

The causes of hospitalization by nursing home residents are clearly complex. Future research needs to take into account the wider context in which decisions to hospitalize (or not) are made. This includes the financial incentives associated with payments to hospitals and general practitioners as well as reimbursement policies for the nursing homes themselves.

## Competing interests

The author declares that she has no competing interests.

## Author information

Toni Ashton, PhD, is Professor of health economics at the University of Auckland, New Zealand. Her main research interests are in the funding and organization of health systems and health care reform, with much of her recent research focusing on various dimensions of health reform in New Zealand. She has co-authored three books, and is on the editorial board of five academic journals, including the Israel Journal of Health Policy Research.

## Commentary on

Wagman S, Rishpon S, Kagan G *et al.*: **Variables correlated with elderly referral from nursing homes to general hospital.***Isr J of Health Policy Res* 2013, 3: 2.

## References

[B1] Wagman S Rishpon S Kagan G Variables correlated with elderly referral from nursing homes to general hospital Isr J of Health Policy Res 2013 3 2 24457020 10.1186/2045-4015-3-2 PMC3902424

[B2] Konetzka RT Spector W Limcangco MR Reducing hospitalizations from long-term care settings Med Care Res Rev 2008 65 1 40 66 17895516 10.1177/1077558707307569

[B3] Carter MW Factors associated with ambulatory care-sensitive hospitalizations among nursing homes residents J Aging Health 2003 15 2 295 331 10.1177/0898264303015002001 12795274

[B4] Young Y Barhydt NR Broderick S Factors associated with potentially preventable hospitalizations in nursing home residents in New York state; A survey of directors of nursing J Am Geriatrics Society 2010 58 5 901 907 10.1111/j.1532-5415.2010.02804.x 20406315

[B5] Ouslander JG Lamb G Perloe M Potentially avoidable hospitalizations in nursing home residents: frequency, causes, and costs J Am Geriatrics Society 2010 58 4 627 635 10.1111/j.1532-5415.2010.02768.x 20398146

[B6] Connolly MJ Broad JB Boyd M Randomised controlled trial of packaged “evidenced” interventions for reducing hospitalisations from residential aged care (RAC): First results from the ARCHUS study European Geriatric Medicine 2013 4 Suppl 1 S171

[B7] Intrator O Grabowski DC Zinn J Schleinitz M Feng Z Miller S Mor V Hospitalization of nursing home residents: the effects of states’ Medicaid payment and bed-hold policies Health Ser Res 2007 42 4 1651 1671 10.1111/j.1475-6773.2006.00670.x PMC1955269 17610442

[B8] Spector WD Limcangco R Williams C Rhodes W Hurd D Potentially avoidable hospitalizations for elderly long-stay residents in nursing homes Med Care 2013 51 8 673 681 10.1097/MLR.0b013e3182984bff 23703648

[B9] Grabowski DC Stewart KA Broderick SM Coots LA Predictors of nursing home hospitalization: a review of the literature Med Care Res Rev 2008 65 1 3 39 18184869 10.1177/1077558707308754

